# Decoding the therapeutic potential mechanism of *Cornus officinalis* in Parkinson’s disease: a network pharmacology insight

**DOI:** 10.3389/fphar.2025.1714796

**Published:** 2025-12-01

**Authors:** Zheng Wu, Jiwei Zhao, Wen Wang, Yuan Dong, Taotao Zhou, Yide Feng, Yalan Deng, Yingmei Feng

**Affiliations:** 1 Beijing Institute of Hepatology, Beijing Youan Hospital, Capital Medical University, Beijing, China; 2 Department of Science and Technology, Beijing Youan hospital, Capital Medical University, Beijing, China; 3 Laboratory for Clinical Medicine, Capital Medical University, Beijing, China; 4 Department of Experimental Animal Laboratory, Xuanwu Hospital Capital Medical University, Beijing, China

**Keywords:** traditional Chinese medicine, mechanism of action, Parkinson’s disease, Cornus officinalis, molecular docking

## Abstract

**Background:**

*Cornus officinalis*, traditionally used for its kidney-tonifying and waist-protecting properties, has recently shown potential therapeutic effects in neurological disorders. However, its mechanisms in Parkinson’s disease (PD) remain unclear.

**Methods:**

This study employed a network pharmacology approach combined with molecular docking to systematically explore the active components of *Cornus officinalis* and their associated signaling pathways in PD.

**Results:**

A total of 11,663 PD-related targets were identified from multiple databases, with 185 overlapping targets obtained from active components of *Cornus officinalis* using SwissTargetPrediction. Protein-protein interaction (PPI) network analysis identified EGFR, TP53, HIF1A, ESR1, PPARG, TNF, HSP90AA1, PTGS2, and SRC as the core targets of *Cornus officinalis* in PD. Gene Ontology (GO) enrichment analysis revealed that *Cornus officinalis* primarily modulates pathways such as MAPK signaling, synaptic function, and lipid metabolism. Kyoto Encyclopedia of Genes and Genomes (KEGG) pathway analysis highlighted the involvement of target genes in the neuro-endocrine-immune network. Molecular docking confirmed strong binding affinities between active components and core targets, with binding energies below −5 kcal/mol. Reactome pathway enrichment analysis further identified the IL-4 and IL-13 signaling pathway as the most significant, suggesting a critical role in regulating immune responses and neuroinflammation. Molecular dynamics simulations further confirmed the stability of the binding between *Cornus officinalis* and the targets.

**Conclusion:**

*Cornus officinalis* exhibits potential therapeutic effects against PD through multi-target and multi-pathway mechanisms, including anti-inflammatory actions, regulation of synaptic function regulation, and metabolic modulation. These findings provide a theoretical foundation for further experimental and clinical validation of *Cornus officinalis* as a promising candidate for PD treatment.

## 1 Introduction

Parkinson’s disease (PD) is a prototypical age-related chronic neurodegenerative disorder, affecting approximately 1% of the global population over the age of 60, with its prevalence significantly increasing with advancing age (Blauwendraat et al., 2019). In China, the number of PD patients is on the rise, driven by population aging and environmental factors (Dorsey et al., 2007). Clinically, PD manifests with a spectrum of motor symptoms (such as tremor, rigidity, bradykinesia, and postural instability) and non-motor symptoms (including sleep disturbances, olfactory dysfunction, autonomic dysfunction, and cognitive and psychiatric disorders). The pathogenesis of PD is multifactorial and complex. Research has indicated that arterial blood pressure variability and related vascular factors may influence cognitive decline in PD patients (Pierzchlińska et al., 2021). Neuroinflammation is recognized as a primary contributor to the degeneration of dopaminergic neurons (Zhang et al., 2023a). Post-degeneration, alterations in neuronal synapses directly or indirectly lead to pathological neural network activity, with synaptic modulation playing a pivotal role in the dysfunction of neural networks in PD (Mallet et al., 2019). Consequently, therapeutic strategies that target only dopamine regulation or electrical signal modulation are insufficient.

Traditional Chinese medicine (TCM), with its multiple active components, offers a promising approach for effective clinical treatment. Shanzhuyu (SZY, *Cornus officinalis* Sieb. Et Zucc.), a TCM from the Rosaceae family, is known for its hemostatic, detoxifying, anti-inflammatory, analgesic, antibacterial, anticancer, and neuroprotective properties. Studies have identified that specific components of *Cornus officinalis*, such as loganin and morroniside, exert protective effects against neurotoxicity in PD by reducing apoptosis, mitochondrial damage, decreased neurite length, and reactive oxygen species (ROS) production (Li et al., 2023; Tseng et al., 2019). Additionally, the use of *Cornus officinalis* -containing compound formulas in treating PD has been shown to protect dopaminergic neurons (Bao et al., 2018; Liu et al., 2024). However, the exact mechanisms underlying these effects remain unclear.

Network pharmacology, a strategy that integrates pharmacology and pharmacodynamics through network construction and topological analysis, leverages virtual computing technologies, high-throughput data, and public databases to combine systematic computation. This approach constructs multi-level networks of disease-phenotype-gene-drug interactions to explore the mechanisms and synergistic effects of compounds in disease treatment (Hopkins, 2008).

**FIGURE 1:**
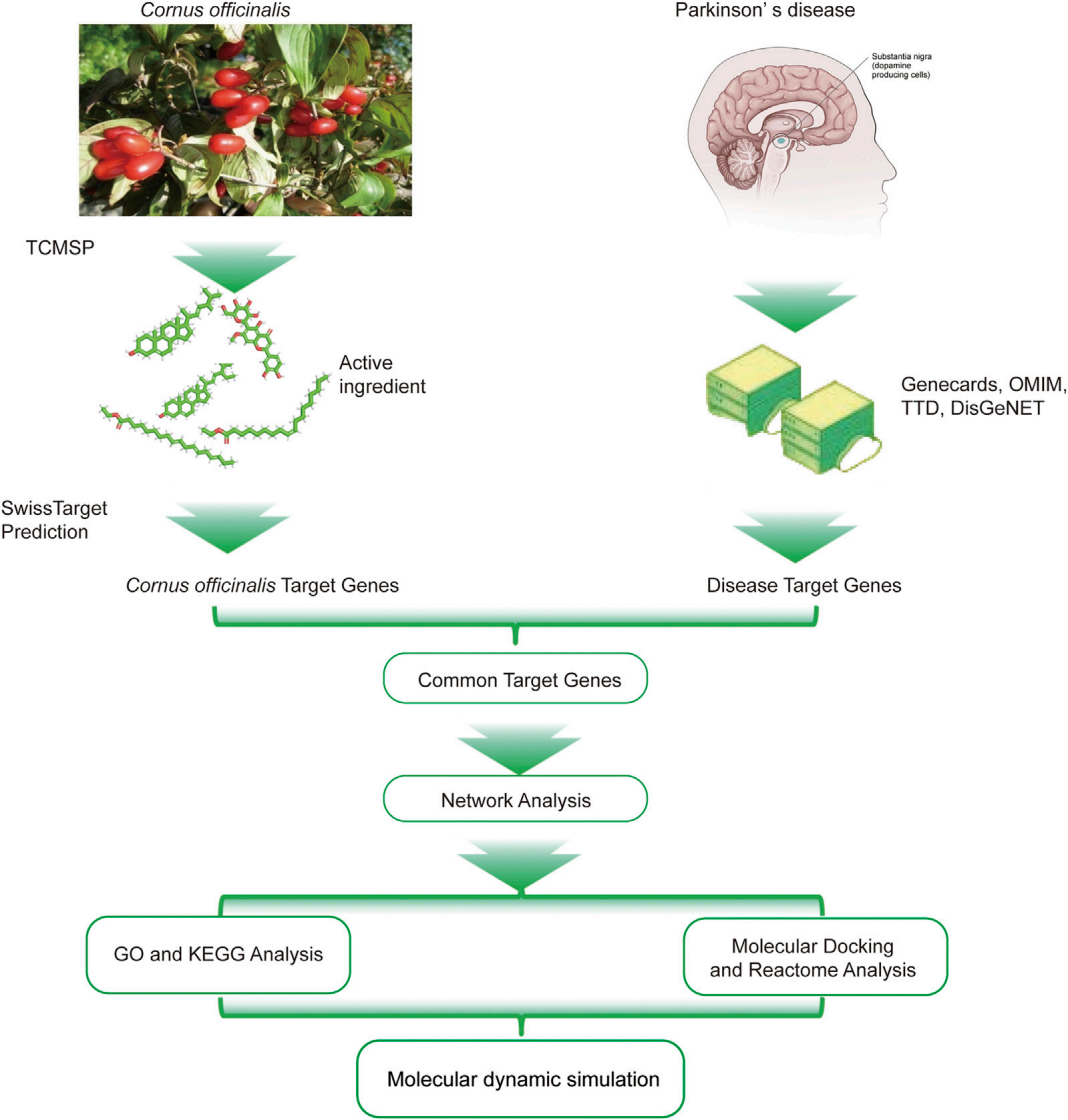
Detailed design flow chart of the current study.

Recent network pharmacology studies have identified the potential of TCM herbs in treating PD (Cai et al., 2025; Qu et al., 2025). Unlike complex TCM formulations, which often consist of multiple herbs with intricate interactions that can be challenging to fully characterize, *Cornus officinalis* offers a well-defined composition and clear mechanisms of action. This clarity not only facilitates scientific research but also enhances its clinical applicability in PD treatment. Additionally, *Cornus officinalis* exhibits a broader spectrum of bioactivities, particularly in neuroprotective effects, which further underscores its therapeutic potential. In this study, we employ network pharmacology to identify potential targets and signaling pathways of *Cornus officinalis* in the treatment of PD, aiming to elucidate its possible mechanisms of action (Figure 1).

## 2 Materials and methods

### 2.1 Acquisition of active compounds and targets

All components of the *Cornus officinalis* were searched from the traditional Chinese medicine systems pharmacology (TCMSP) database (http://tcmspw.com/), which is unique but has comprehensive data and functions (Ru et al., 2014) (accessed on March 28, 2025). It can provide systematic information on the Absorption Distribution Metabolism and Excretion (ADME) characteristics of a drug with potential biological function such as, oral bioavailability (OB) and drug-likeness (DL). The drug screening criteria of OB ≥30% and DL ≥0.18%, as suggested by the TCMSP database, were set as the cut-off values for evaluating the bioactive components (Mokhtari et al., 2025; Wang et al., 2019b).

### 2.2 Prediction of targets for active compounds

To comprehensively predict the targets of *Cornus officinalis* active compounds, in addition to the related targets provided by the TCMSP database, we utilized the SwissTargetPrediction platform (2019 version) (http://swisstargetprediction.ch/), an advanced computational tool designed to predict the most likely protein targets of small molecules (Daina et al., 2019) (accessed on March 28, 2025). This platform identifies target genes based on the SMILES (Simplified Molecular Input Line Entry System) codes of the active compounds, selecting the top 20 target genes for each compound. The target genes from both databases were consolidated, duplicates were removed, and the relationships between each compound and its corresponding target genes were visualized in Cytoscape software (3.10.3 version).

### 2.3 Screening of PD-related targets

Four databases—GeneCards (https://www.genecards.org/) that provides a comprehensive and authoritative summary to enable researchers to effectively navigate and inter-relate the vast universe of human genes and diseases (Stelzer et al., 2016), Online Mendelian Inheritance in Man (OMIM) (http://www.omim.org) considered comprehensive, authoritative compendiums of human genes and genetic phenotypes, which are widely used in analyzing drug components and disease-related targets, Therapeutic Target Database (TTD) (https://db.idrblab.net/ttd/) is a database that furnishes information on acknowledged and explored therapeutic proteins and targeted diseases, nucleic acid targets and pathways as well as the corresponding drugs directed at each of these targets (Zhou et al., 2024), and DisGeNET (http://www.disgenet.org/) is a discovery platform containing one of the largest publicly available collections of genes and variants associated with human diseases (Piñero et al., 2016)—were used to collect target genes associated with “Parkinson’s disease” (accessed on April 1, 2025). Targets with a Score_gda ≥0.7 in DisGeNET were included (Wang et al., 2023). After merging and removing duplicates, the final set of PD-related target genes was obtained.

### 2.4 Clustering of *Cornus officinalis* and PD related target genes and screening key genes

To identify the overlapping targets between *Cornus officinalis* active components and PD-related genes, we used Venny 2.1.0 (https://bioinfogp.cnb.csic.es/tools/venny/index.html), a user-friendly tool for visualizing intersections between datasets (accessed on April 11, 2025), was analyzed to intersect the *Cornus officinalis* active component targets with the PD targets. Target genes were obtained using the Venny tool. The overlapping target genes were then imported into STRING database V11 (accessed on April 11, 2025), a widely used platform for constructing functional protein association networks (Szklarczyk et al., 2018). STRING integrates known and predicted protein interactions from multiple sources, enabling comprehensive analysis of protein relationships and interactions. The medium confidence level was set to greater than 0.4, for the species “*Homo sapiens*” (An et al., 2022). The protein-protein interaction (PPI) network construction data were exported from STRING and was imported into Cytoscape (3.10.3 version).

To identify significant modules within the PPI network, the MCODE algorithm was applied with the following parameters: degree cutoff = 2, node score cutoff = 0.2, k-core = 2, and max depth = 100 (Zijie et al., 2022). Modules with an MCODE score ≥4 were considered significant and further analyzed for their biological relevance to PD. Hub genes within these modules were identified using the Maximal Clique Centrality (MCC) algorithm, with genes exhibiting the highest MCC scores considered key regulators of the module’s biological function (Chin et al., 2014).

### 2.5 Gene Ontology (GO) and Kyoto Encyclopedia of Genes and Genomes (KEGG) pathway enrichment analyses

The input molecular list was first converted to compatible IDs using org. Hs.eg.db, followed by GO and KEGG enrichment analysis with clusterProfiler, with significance set at p < 0.05. The top 20 enriched terms in GO biological processes (BP), cellular components (CC), molecular functions (MF), and KEGG pathways were selected and visualized for interpretation.

### 2.6 Molecular docking

The 3D structures of the active components of *Cornus officinalis* in SDF format were retrieved from PubChem. The 3D structures of nine target proteins in PDB format were obtained from the Protein Data Bank (https://www.rcsb.org/)(accessed on April 17, 2025). Subsequently, PyMOL was used to remove ligands and water molecules from the target proteins. After this preprocessing step, CB-Dock (http://cao.labshare.cn/cb-dock/), which employs cavity detection, was utilized for blind docking. It can automatically locate potential binding sites within a protein, compute their center and size, and tailor the docking box size to the query ligands. Subsequently, molecular docking was conducted with a popular docking program, AutoDock Vina (Liu et al., 2022b; Wang et al., 2022a) (accessed on April 23, 2025). From the docking results, the conformation with the highest absolute score was selected. The docking results were visualized using PyMOL (https://pymol.org/2/), where hydrogen bonds and binding sites were analyzed. Additionally, the software Discovery Studio (4.5 Visualizer) was utilized to generate high-quality 2D representations of the small molecules and target proteins (Jannat et al., 2022).

### 2.7 Reactome pathway enrichment analysis

The Reactome analysis data were exported from the STRING platform using nine targets validated by molecular docking, with the species set to “*Homo sapiens*” and a significance threshold of FDR <0.05. The results were filtered by Top 10 and then imported into lollipop plots for visualization.

### 2.8 Molecular dynamic simulation

The molecular dynamics simulations were carried out with Desmond/Maestro noncommercial (Desmond molecular dynamics system, version 2022.1. Research, New York, NY.) as a molecular dynamic’s software (Bowers et al., 2006). TIP3P water molecules were added to the systems, which were then neutralized by 0.15 M NaCl solution to approximate physiological saline conditions. After energy minimization and relaxation of the system, the production simulation was performed for 100 ns for all ligand–protein complexes in an isothermal-isobaric ensemble at 300 K and 1 bar. The temperature of 300 K was chosen as it reflects standard room temperature (∼27 °C) and is relevant for biological processes such as enzyme activity and protein folding. The pressure of 1 bar represents standard atmospheric pressure, and the NPT ensemble allows volume fluctuations to maintain constant pressure, simulating realistic solution conditions. Trajectory coordinates were recorded every 100 ps. The molecular dynamics analysis was performed using Simulation Interaction Diagram from Desmond. Structural stability and flexibility of the complexes were evaluated through Root Mean Square Deviation (RMSD) and Root Mean Square Fluctuation (RMSF) analyses, radius of gyration (rGyr) and solvent-accessible surface area (SASA) of the complexes (Deng et al., 2025).

## 3 Results

### 3.1 Active components of *Cornus officinalis* and their targets

**FIGURE 2:**
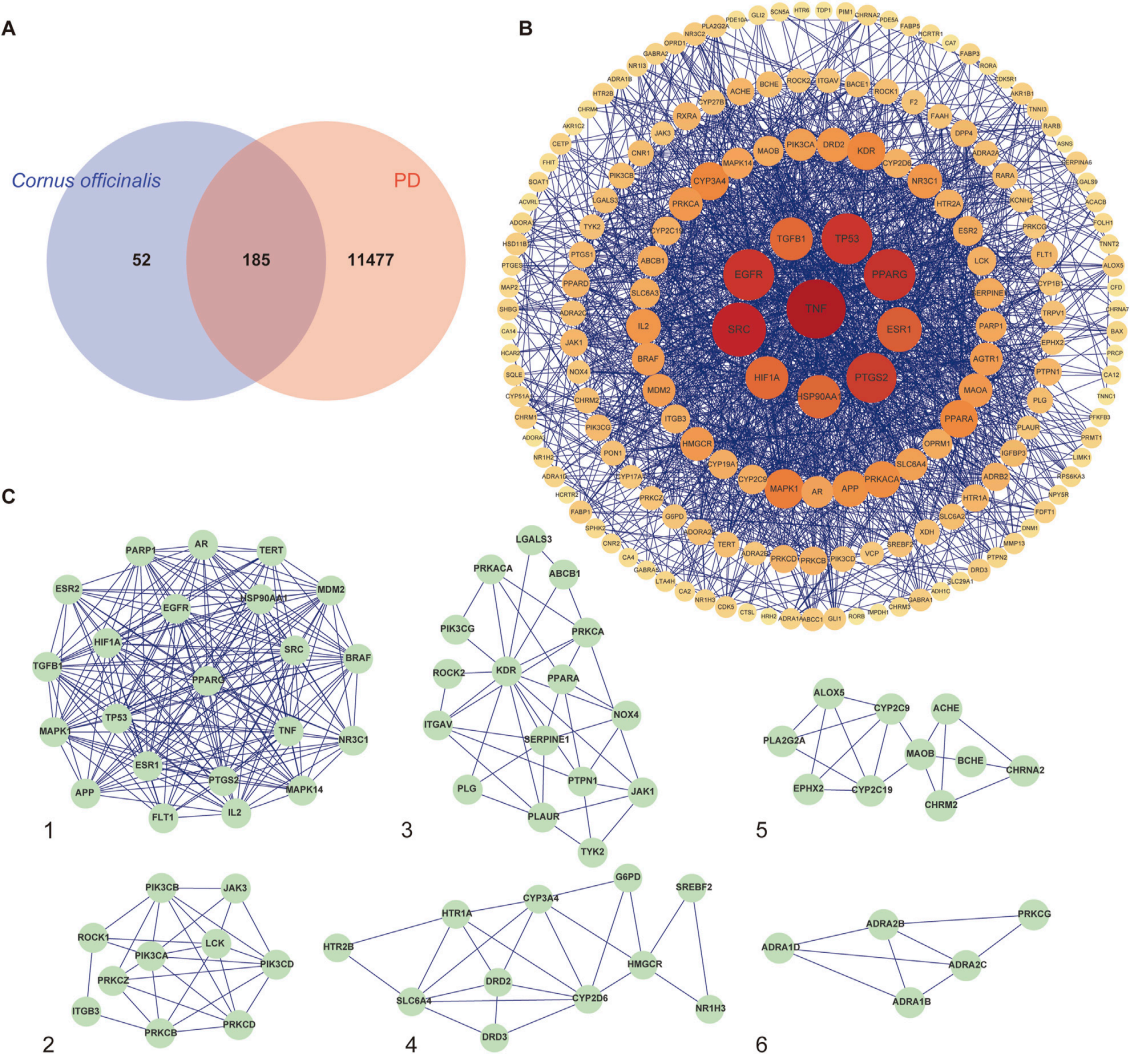
Active compounds and targets of *Cornus officinalis*. **(A)** Venny diagram of active compound of *Cornus officinalis* and PD common targets. **(B)** *Cornus officinalis* and PD common target network. **(C)** MCODE analysis results of the PPI network.

Potential active components of *Cornus officinalis* were screened from the aforementioned databases, and their basic information is presented in Table 1. A total of 11662 PD-related targets were identified from the GeneCards, DisGeNET, TTD, and OMIM databases. Venn diagram analysis revealed 185 overlapping targets (Supplementary Table S1), which were considered common targets (Figure 2A).

**TABLE 1:** 20 compounds from *Cornus officinalis* screening condition OB ≥30%, DL ≥0.18%.

Molecule name	Chemical structure	OB(30%)	DL (0.18%)
Mandenol	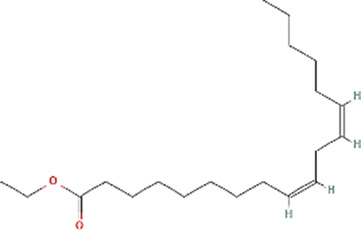	42	0.19
Ethyl linolenate	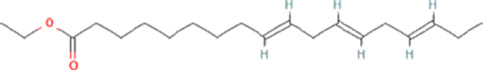	46.1	0.2
Poriferast-5-en-3beta-ol	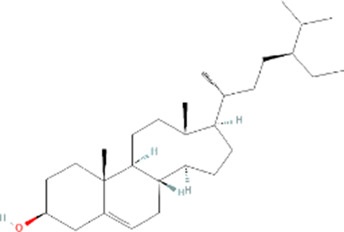	36.91	0.75
Diop	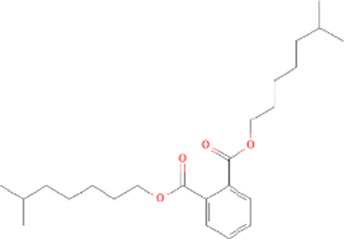	43.59	0.39
Ethyl oleate (NF)	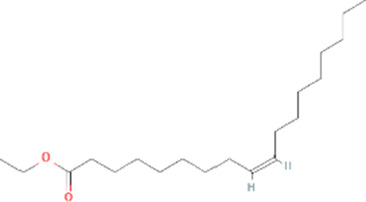	32.4	0.19
Leucanthoside	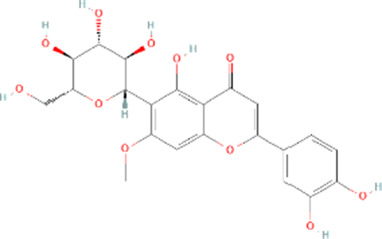	32.12	0.78
Beta-sitosterol	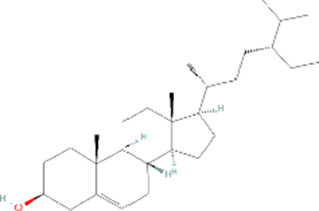	36.91	0.75
Sitosterol	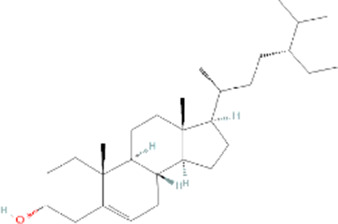	36.91	0.75
Stigmasterol	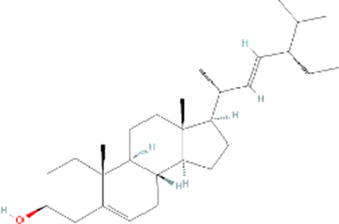	43.83	0.76
Malkangunin	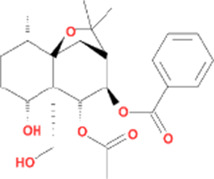	57.71	0.63
2,6,10,14,18-Pentamethylicosa-2,6,10,14,18-pentaene	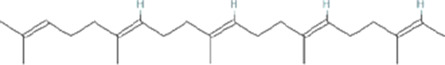	33.4	0.24
3,4-Dehydrolycopen-16-al	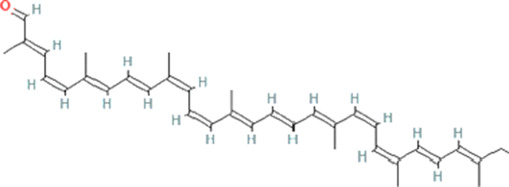	46.64	0.49
3,6-Digalloylglucose	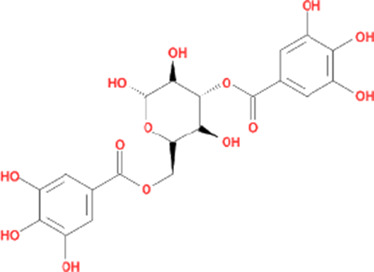	31.42	0.66
Cornudentanone	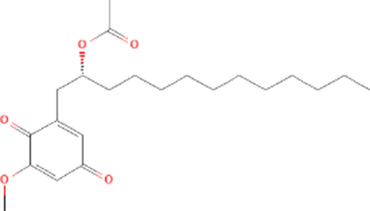	39.66	0.33
Hydroxygenkwanin	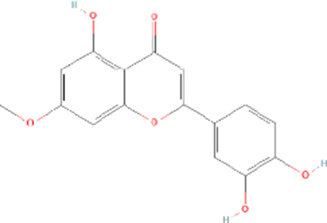	36.47	0.27
Telocinobufagin	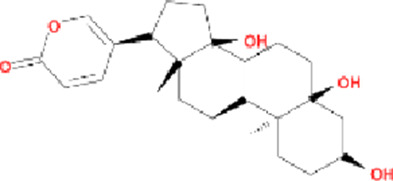	69.99	0.79
Tetrahydroalstonine	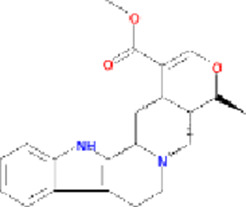	32.42	0.81
Gallic acid-3-O-(6′-O-galloyl)-glucoside	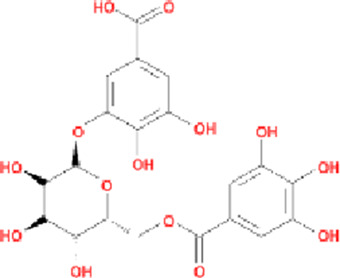	30.25	0.67
Gemin D	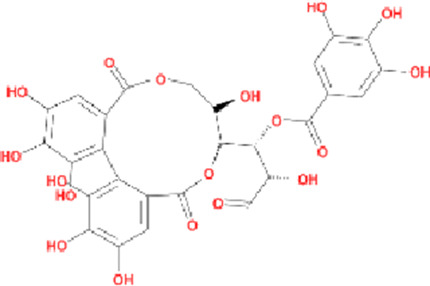	68.83	0.56
Lanosta-8,24-dien-3-ol,3-acetate	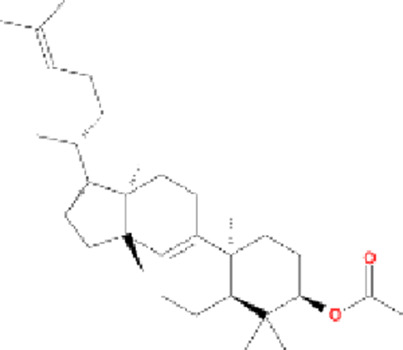	44.3	0.82

OB, oral bioavailability; DL, drug-likeness.

### 3.2 Common target network

The 185 common targets were imported into the STRING database to obtain 181 protein-protein interactions (PPIs). The network of active components and disease targets was constructed using Cytoscape software (Figure 2B). Network analysis showed 181 nodes and 1,700 edges, with an average node degree of 18.785 and an average local clustering coefficient of 0.481.

**FIGURE 3:**
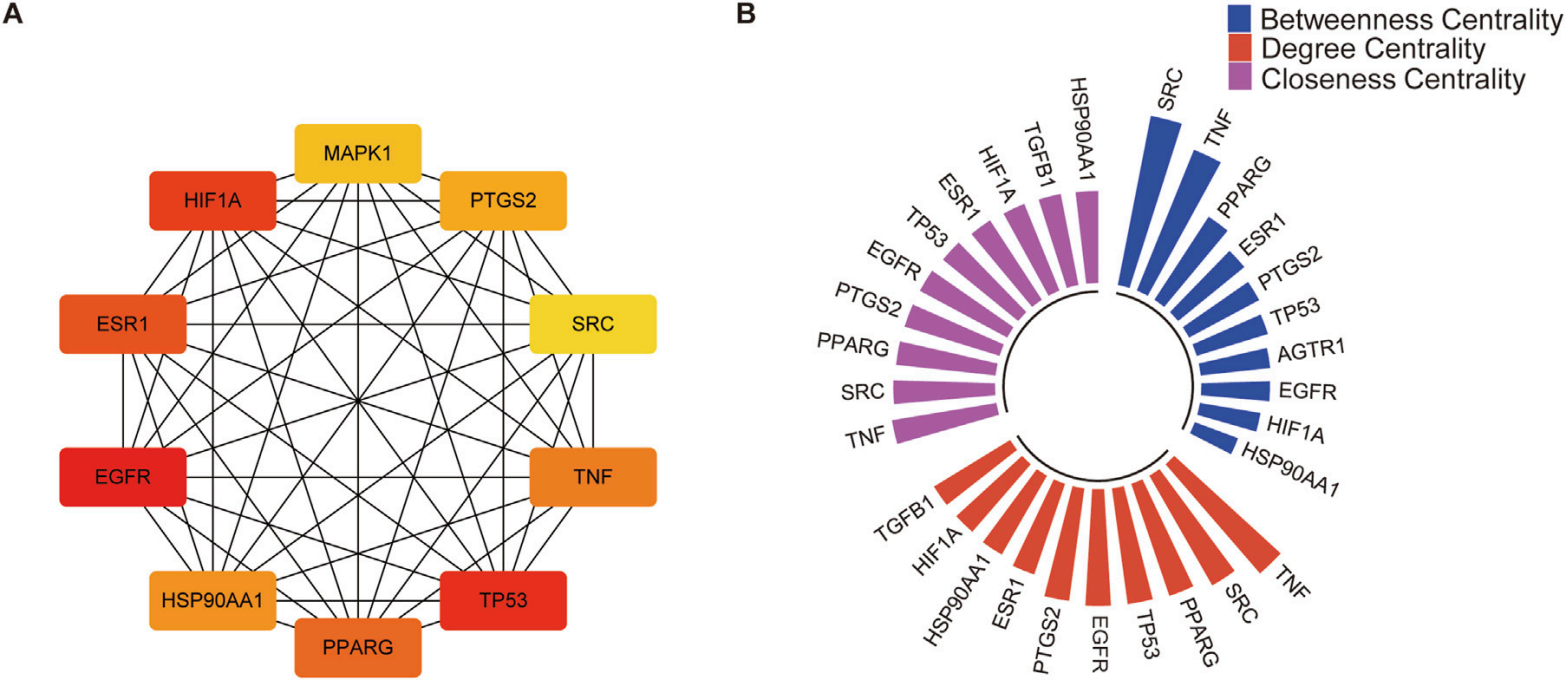
Identification of key targets of *Cornus officinalis* for PD treatment using network centrality metrics. **(A)** Identification of the top 10 hub genes in the network by MCC algorithm. **(B)** The degree of connectivity, closeness centrality, and betweenness centrality of the top 10 targets.

The PPI network was analyzed using the MCODE plugin in Cytoscape software, which identified six significant clusters (MCODE score ≥4) (Figure 2C; Supplementary Table S2). Additionally, the top 10 hub genes were determined using the CytoHubba plugin, which implements the MCC algorithm. The MCC algorithm identifies central nodes by detecting all maximal cliques in the network, evaluating the participation of each node in these cliques, and assigning centrality scores based on their level of involvement. The identified hub genes include Epidermal Growth Factor Receptor (EGFR), Tumor Protein p53 (TP53), Hypoxia-Inducible Factor 1-Alpha (HIF1A), Estrogen Receptor Alpha (ESR1), Peroxisome Proliferator-Activated Receptor Gamma (PPARG), Tumor Necrosis Factor (TNF), Heat Shock Protein 90 Alpha Family Class A Member 1 (HSP90AA1), Prostaglandin-Endoperoxide Synthase 2 (PTGS2), Mitogen-Activated Protein Kinase 1 (MAPK1), and SRC Proto-Oncogene (SRC) (Figure 3A).

The top 10 genes were identified based on three centrality measures: degree, betweenness, and closeness (Figure 3B). Degree centrality reflects the number of direct connections a gene has within the network, indicating its local influence (Schwartz et al., 2025). Betweenness centrality identifies genes that serve as bridges between different parts of the network, highlighting their role in facilitating communication across the network. Closeness centrality measures how quickly a gene can interact with all other genes in the network, emphasizing its global accessibility and efficiency in information transfer (Özgür et al., 2008). Based on these analyses, nine central targets of *Cornus officinalis* against PD were identified: EGFR, TP53, HIF1A, ESR1, PPARG, TNF, SRC, HSP90AA1, and PTGS2.

**FIGURE 4:**
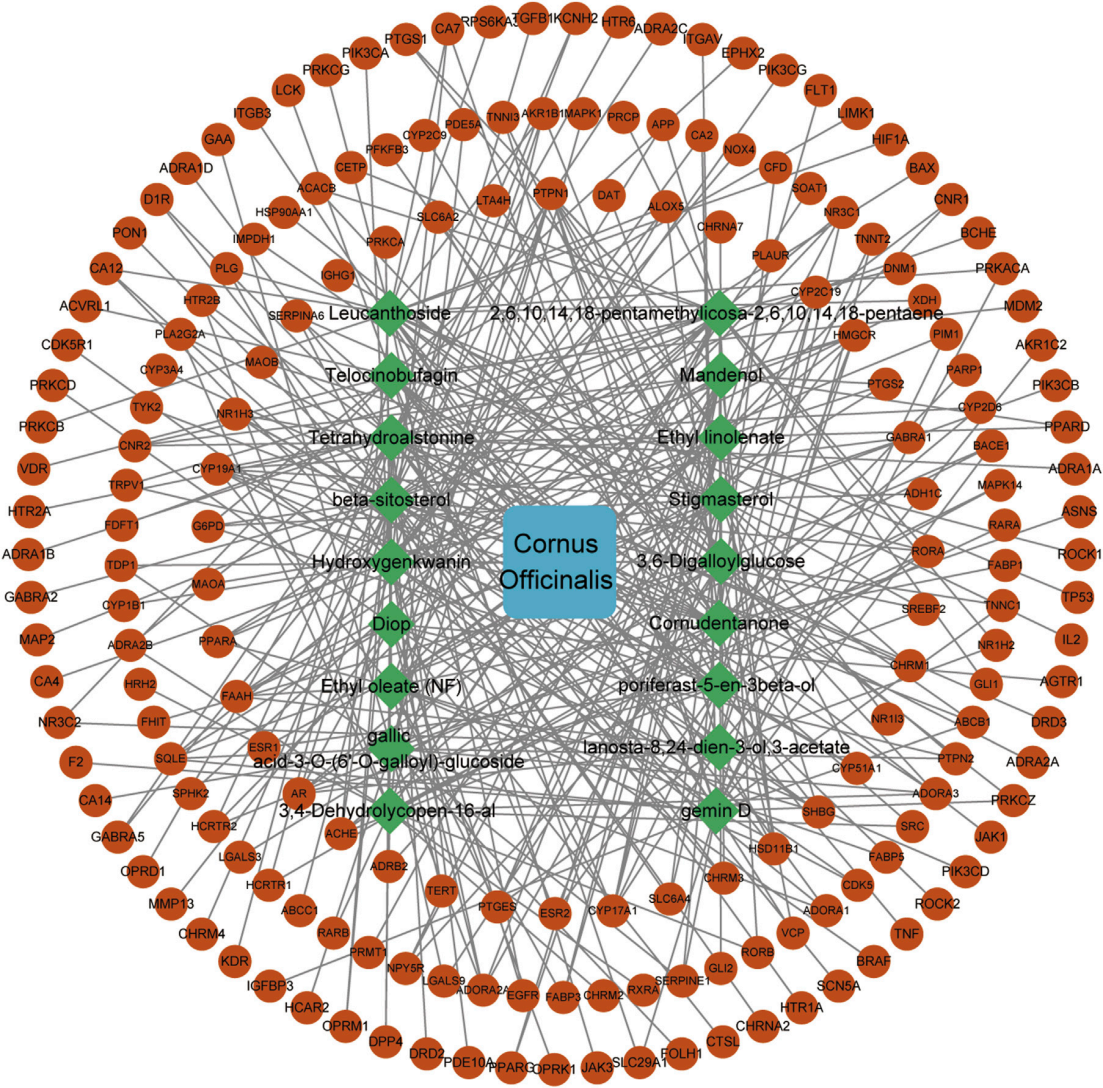
*Cornus officinalis* active ingredients-potential targets network.

Non-target and duplicate active compounds were removed, resulting in 17 compounds. The “active compounds-disease targets” network was constructed using Cytoscape 3.10.3, incorporating overlapping targets and related active components (Figure 4).

### 3.3 GO and KEGG pathway enrichment analysis of core targets

**FIGURE 5:**
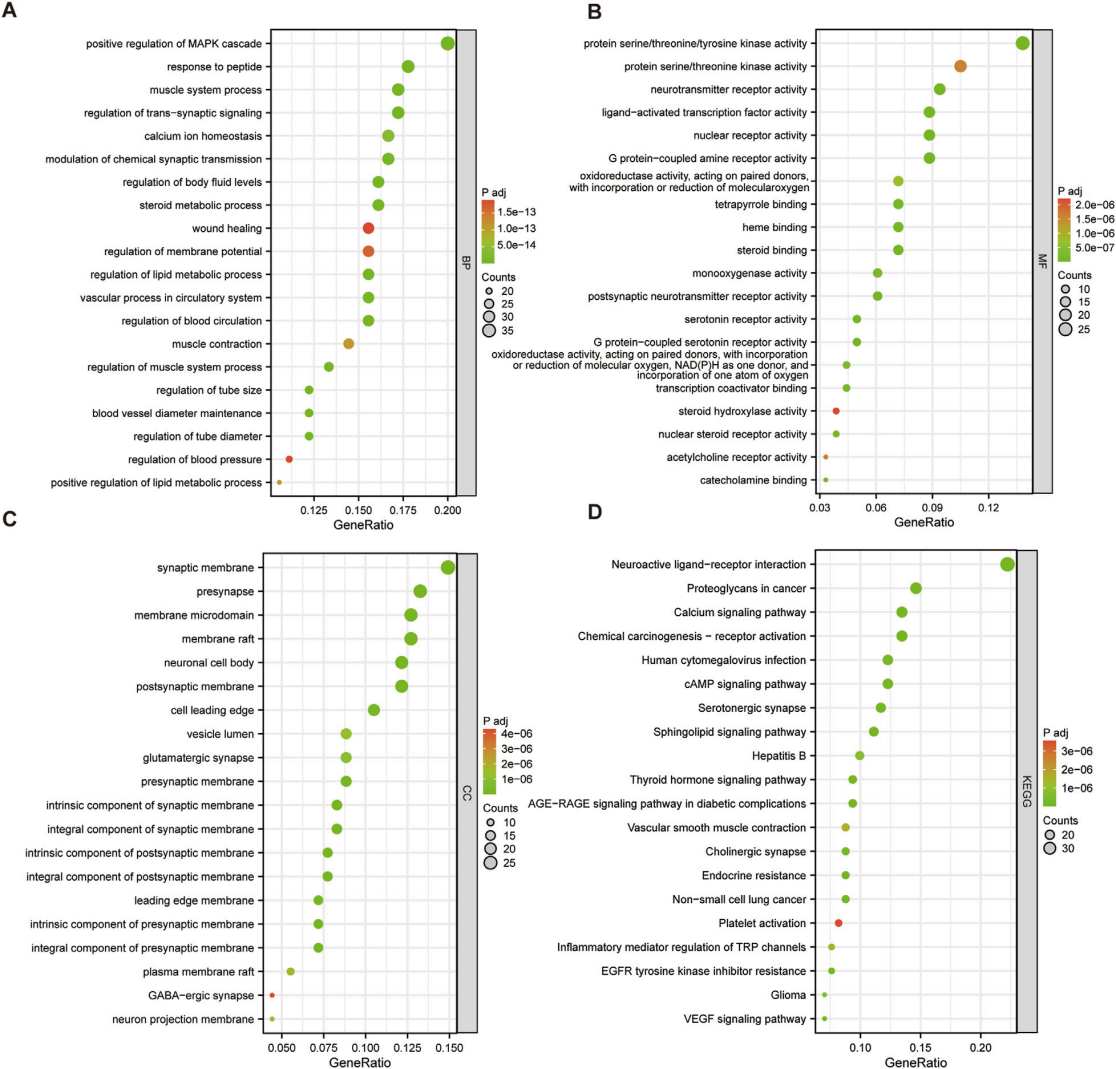
GO and KEGG pathway enrichment analysis of *Cornus officinalis* targets for the treatment of PD. **(A)** GO Dotplots for biological process (BP). **(B)** GO Dotplots for molecular function (MF). **(C)** GO Dotplots for cellular component (CC). **(D)** Dotplot illustrating the findings of the KEGG enrichment analysis.

Based on the results of GO and KEGG enrichment analysis, we can further explore the potential mechanisms and significance of *Cornus officinalis* in the treatment of PD (Supplementary Table S3). The BP associated with *Cornus officinalis* in PD treatment primarily involved regulating MAPK signaling, blood circulation, vascular function, and lipid metabolism (Figure 5A). These findings suggest that *Cornus officinalis* may exert neuroprotective effects by regulating cellular signaling and metabolic homeostasis, both of which are critical to PD pathophysiology.

MF were mainly related to transmembrane signal transduction, kinase phosphorylation, transcriptional regulation, and oxidative metabolism (Figure 5B). These functions are closely associated with neurotransmission, hormonal responses, and energy metabolism, further supporting the therapeutic potential of *Cornus officinalis* in PD.

CC analysis revealed enrichment in synaptic structures and neuronal functions, including presynaptic/postsynaptic membranes, synaptic membranes, and neuronal cell bodies (Figure 5C). This suggests that *Cornus officinalis* may play a role in synaptic plasticity and neuronal signaling, which are key mechanisms in PD progression.

KEGG pathway analysis highlighted the pivotal role of target genes in the neuro-endocrine-immune network, particularly in transmembrane signal transduction (GPCRs, ion channels), kinase phosphorylation (EGFR, VEGFR) (Figure 5D). The active components of *Cornus officinalis* may exert neuroprotective effects by modulating synaptic function, inhibiting neuroinflammation, and improving metabolic disorders.

### 3.4 Molecular docking and reactome analysis

**FIGURE 6:**
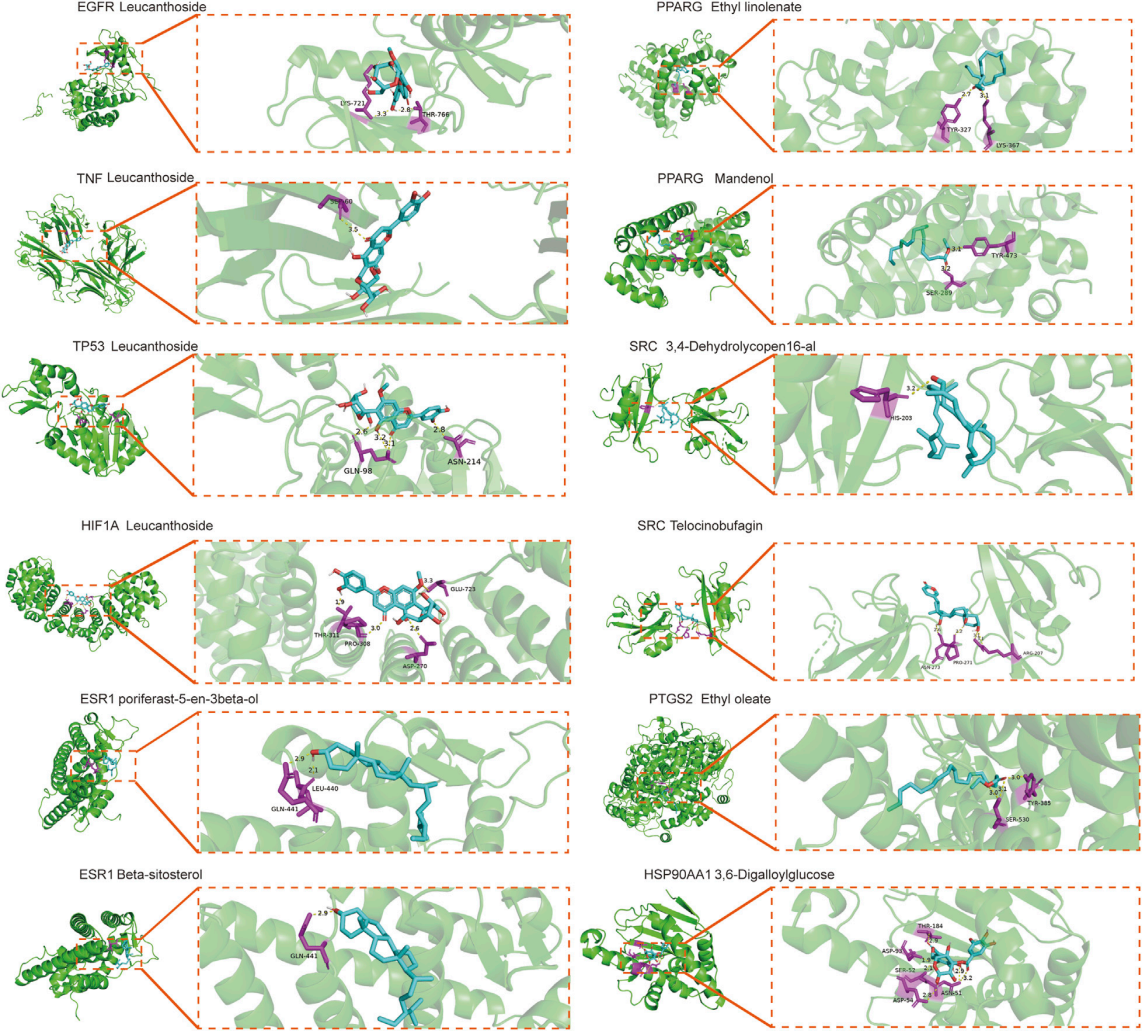
Diagrammatic 3D illustrated the molecular docking model.

**FIGURE 7:**
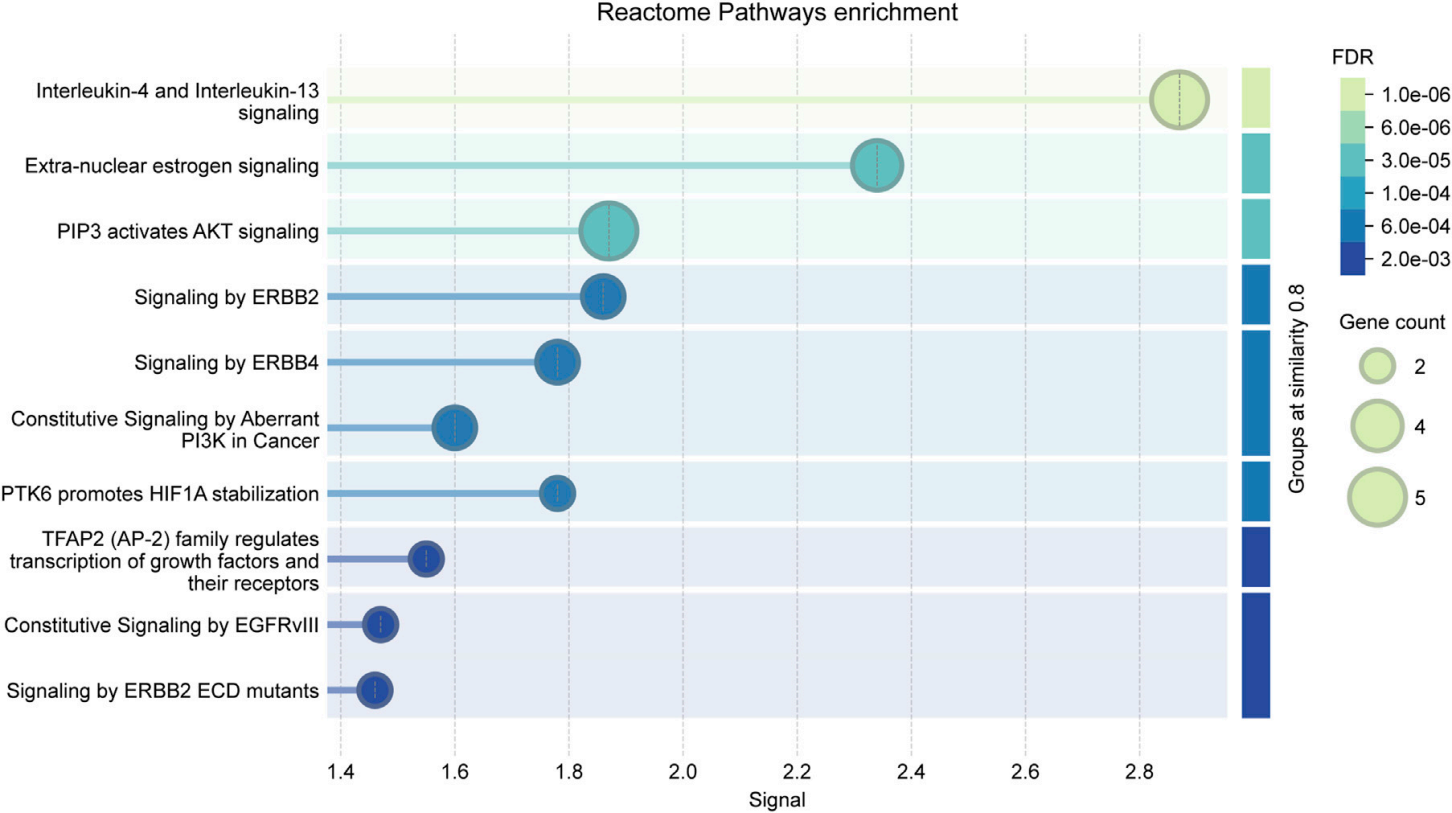
Lollipop plots displaying Reactome pathway enrichment of 9 core targets.

Molecular docking was performed to evaluate the binding affinity between *Cornus officinalis* active components and target proteins (Table 2). A binding energy of <−5 kcal/mol was considered strong binding, with lower values indicating stronger docking. The results demonstrated high affinity between the core targets and their corresponding compounds (Figure 6; Supplementary Figure S1; Table 3). Reactome pathway enrichment analysis further supported the importance of the IL-4/IL-13 signaling pathway, which was ranked as the top pathway associated with the core targets (Figure 7; Supplementary Table S4). This pathway is known to regulate regulating immune responses, particularly in anti-inflammatory and allergic reactions, suggesting that *Cornus officinalis* may alleviate neuroinflammation in PD.

**TABLE 2:** PDB ID and resolution of 9 targets for *Cornus officinalis* against PD.

Protein name	PDB ID	Resolution
EGFR	1M17	2.60 Å
TNF	2AZ5	2.10 Å
TP53	1YC5	1.40 Å
HIF1A	8HE3	1.90 Å
ESR1	1XP1	1.80 Å
PPARG	2HFP	2.00 Å
SRC	1NZL	1.90 Å
PTGS2	3NT1	1.73 Å
HSP90AA1	3O0I	1.47 Å

**TABLE 3:** Molecular docking energy scoring results of *Cornus officinalis* binding to targets (kcal/mol).

Receptor name	Ligand name	Score (kcal/mol)
EGFR	Leucanthoside	−8.1
TNF	Leucanthoside	−9.3
TP53	Leucanthoside	−8.0
HIF1A	Leucanthoside	−7.4
ESR1	Poriferast-5-en-3beta-ol	−7.7
ESR1	Beta-sitosterol	−7.1
PPARG	Ethyl linolenate	−6.4
PPARG	Mandenol	−6.5
SRC	3,4-Dehydrolycopen-16-al	−6.9
SRC	Telocinobufagin	−8.1
PTGS2	Ethyl oleate	−6.4
HSP90AA1	3,6-Digalloylglucose	−9.1

### 3.5 Molecular dynamics simulation of core targets

**FIGURE 8:**
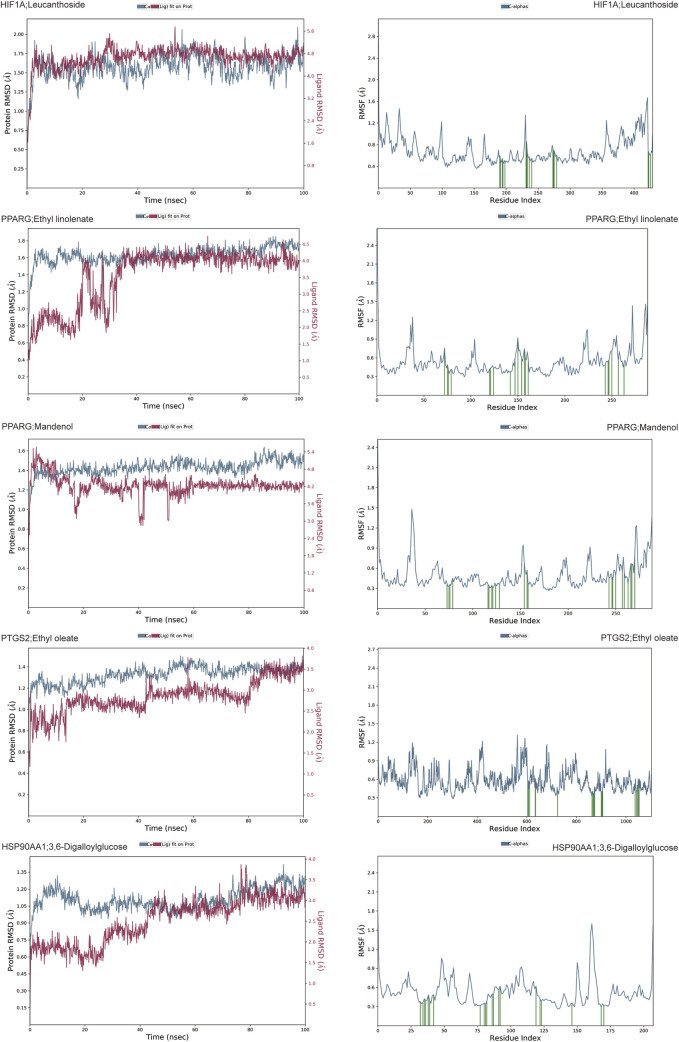
RMSD and RMSF analysis of HIF1A-Leucanthoside, PPARG-Ethyl linolenate, PPARG-Mandenol, PTGS2-Ethyl oleate, and HSP90AA1-3,6-Digalloylglucose complexes.

To further assess the binding affinity of the active components of *Cornus officinalis* to the core targets, molecular dynamics simulations were performed. RMSD, a robust metric for evaluating the conformational stability of protein-ligand complexes, was utilized, with lower RMSD values indicating greater stability. The results revealed that complexes involving HIF1A-Leucanthoside, PPARG-Ethyl linolenate, PPARG-Mandenol, HSP90AA1-3,6-Digalloylglucose, and PTGS2-Ethyl oleate exhibited superior stability (Figure 8; Supplementary Figure S2).

RMSF was employed to characterize the fluctuations of individual amino acid residues during the simulation. RMSF analysis further confirmed the stability of these complexes, with lower fluctuations observed in key amino acid residues (Figure 8; Supplementary Figures S3,S4). Further analysis revealed that rGyr and SASA remained stable throughout the simulation, indicating minimal conformational changes in the complexes (Supplementary Figure S5), indicating that the complexes underwent conformational changes without significant contraction or expansion. In conclusion, the complexes exhibited stable binding, with the small molecules effectively interacting with the target proteins. Among them, HIF1A-Leucanthoside, PPARG-Ethyl linolenate, PPARG-Mandenol, HSP90AA1-3,6-Digalloylglucose, and PTGS2-Ethyl oleate demonstrated superior stability.

## 4 Discussion

PD is a complex neurodegenerative disorder characterized by the degeneration of dopaminergic neurons, the aggregation of α-synuclein (α-syn), and neuroinflammation (Blauwendraat et al., 2019). In this study, TCM has emerged as a promising avenue for PD treatment, as its active components exhibit multifaceted mechanisms of action, including anti-apoptosis, anti-inflammation, antioxidant stress, restoration of mitochondrial function, modulation of autophagy, and regulation of neurotransmitters (Chen et al., 2022). Network pharmacology, a systems biology-based approach, has become a powerful tool for elucidating the complex mechanisms of TCM. In this study, we employed network pharmacology combined with molecular docking analysis to explore the molecular mechanisms underlying the anti-PD effects of *Cornus officinalis*. Our findings identified several active components of *Cornus officinalis* that may contribute to its anti-PD effects, including Poriferast-5-en-3beta-ol, Beta-sitosterol, Ethyl linolenate, Mandenol, 3,4-Dehydrolycopen-16-al, Telocinobufagin, Ethyl oleate, and 3,6-Digalloylglucose. These compounds are likely to exert their therapeutic effects through interactions with key molecular targets. Furthermore, our analysis revealed that EGFR, TNF, TP53, HIF1A, ESR1, PPARG, SRC, PTGS2, and HSP90AA1 are potential therapeutic targets of *Cornus officinalis* in PD treatment.

Leucanthoside, a novel allose-containing triterpenoid saponin, emerged as a primary active compound in our study, targeting EGFR, TNF, TP53, and HIF1A. While limited research has been conducted on the biological activities of Leucanthoside, structurally similar compounds such as Swertiajaponin have demonstrated neuroprotective effects, suggesting potential benefits in neurodegenerative diseases (Bellavite, 2023; Moon et al., 2018). Based on our computational analysis and literature correlations, we hypothesize that Leucanthoside may exert its effects through the modulation of critical signaling pathways that regulate cell growth, survival, and anti-oxidative stress. This hypothesis is supported by the known roles of its predicted targets (EGFR, TNF, TP53, and HIF1A) in PD pathophysiology, as described below. TNF-α, a pro-inflammatory cytokine, is crucial in host defense mechanisms. In PD, TNF-α is known to activate microglia, contributing to progressive neuronal loss (Liu et al., 2022a). Furthermore, elevated peripheral blood TNF-α levels have been correlated with higher UPDRS scores in PD patients (Xiromerisiou et al., 2022). Upregulation of TNF enhances the susceptibility of cells in the central nervous system to apoptosis and cell death, thereby exacerbating neuroinflammation (Yuan et al., 2019). TP53, a central regulator of processes such as the cell cycle and apoptosis, has been implicated in PD through its involvement in mitochondrial dysfunction, ROS production, abnormal protein aggregation, and impaired autophagy (Luo et al., 2022). The hypoxia/HIF-1α signaling pathway is also implicated in mitochondrial dysfunction, oxidative stress, and defective protein degradation in PD (Leston et al., 2021). Additionally, the PD-associated protein α-syn induces neuroinflammation through the IL6ST-AS/STAT3/HIF-1α axis (Lin et al., 2023). While our study predicts that Leucanthoside may target TNF, TP53 and HIF1A, further experimental validation is required to confirm this interaction and its therapeutic implications.

Other potential targets of *Cornus officinalis* in PD treatment include ESR1, PPARG, SRC, PTGS2, and HSP90AA1, which are associated with the amelioration of inflammatory responses (Semenova et al., 2023; Smajic et al., 2022; Titus et al., 2024; Yang et al., 2020). Further research is essential to fully elucidate the effects of *Cornus officinalis* and to explore its clinical applications.

*Cornus officinalis* has traditionally been used for its kidney-tonifying and waist-protecting effects. However, increasing research has revealed its neuroprotective potential in various brain disorders. *Cornus officinalis* exhibits neuroprotective effects against H2O2-induced cytotoxicity in SH-SY5Y cells and stress-induced hippocampal deficits in rats. Cornel iridoid glycosides (CIGs) from *Cornus officinalis* inhibit oxidative stress and neurotransmitter hydrolases, increasing acetylcholine and monoamine neurotransmitters, thereby ameliorating cognitive impairments in Alzheimer’s disease (Wang et al., 2022b). Loganin, another component of *Cornus officinalis*, alleviates gap junction dysfunction in astrocytes of the prefrontal cortex and hippocampus in depression models via the GSK-3β/β-catenin signaling pathway. It also increases serotonin and dopamine levels, mitigates hypothalamic-pituitary-adrenal axis dysfunction, and enhances BDNF expression to improve stress-related depressive symptoms (Guo et al., 2023; Wang et al., 2025). Loganin attenuates inflammation, oxidative stress, and apoptosis through the JAK2/STAT3 pathway. CIGs reduce cerebral ischemia-reperfusion injury by inhibiting microglial activation and neuroinflammation via the TLR4/MyD88/NF-κB signaling pathway. Additionally, they regulate polarized aquaporin 4 to alleviate post-reperfusion cerebral edema (Guo et al., 2025; Wang et al., 2024). CIGs mitigate neuroinflammation in autoimmune encephalitis and traumatic brain injury by inhibiting the JAK/STAT and NF-κB/STAT3 pathways, while protecting against white matter lesions in rat models of cerebral ischemia through activation of the BDNF/Neuregulin-1 pathway (Qu et al., 2019; Wang et al., 2019a; Zheng et al., 2019). Furthermore, components of *Cornus officinalis* have been shown to promote neurogenesis and angiogenesis following cerebral ischemia-reperfusion (Liu et al., 2016; Wang et al., 2010).

Through GO analysis of intersecting target genes, it was found that *Cornus officinalis* primarily acts on the MAPK signaling pathway in the treatment of PD. In PD, the MAPK cascade is upregulated, including extracellular signal-regulated kinase, c-Jun N-terminal kinase, and p38. Their abnormal activation can lead to oxidative stress, impaired ROS/NO balance, microglial activation, and chronic inflammation (Jha et al., 2015; Obergasteiger et al., 2018). Pathological activation of p38 MAPK induces serine 131 phosphorylation, resulting in mitochondrial dysfunction and neuronal degeneration (Chen et al., 2018). Enzymatic cleavage of α-syn and tau proteins generates α-SynN103 and tauN368, which increase p38 MAPK activity, affecting synaptic membrane structure and anterograde axonal transport (Yang et al., 2025). Mitochondria at presynaptic terminals respond to changes in intracellular Ca2+ during action potentials, thereby influencing neurotransmitter release and synaptic vesicle cycling. GO enrichment analysis revealed that *Cornus officinalis* plays a role in neuronal cell bodies, synaptic structures, and synaptic membrane integrity in the MF category, and regulates membrane signaling in the CC category, suggesting its therapeutic mechanisms in PD. *Cornus officinalis* ameliorates neurotoxicity by regulating mitochondrial autophagy in PD neurons, and autophagy may be induced by p38 MAPK, contributing to neurodegeneration (Zhou et al., 2025). Furthermore, *Cornus officinalis* improves blood circulation and provides neuroprotection through the remodeling of the neurovascular unit, an effect previously observed in stroke (Liu et al., 2016).

The neural, immune, and endocrine communication pathways between the gastrointestinal tract and the central nervous system are a hot topic in PD pathology (Zhang et al., 2023b; Zheng et al., 2023). KEGG enrichment analysis revealed that *Cornus officinalis* targets pathways such as neuroactive ligand-receptor interaction, AGE-RAGE signaling pathway in diabetic complications, thyroid hormone signaling pathway, and endocrine resistance, which may represent breakthroughs in its therapeutic effects on PD through the neuro-endocrine-immune network.

The Reactome Analyze Data tool was utilized to validate the potential pathways involving nine key genes. Reactome analysis revealed that the IL-4 and IL-13 signaling pathways encompassed potential targets: HSP90AA1, PTGS2, HIF1A, TP53, and TNF, all of which are regulated by STAT3. IL-4 and IL-13 are Th2-type cytokines known for their anti-inflammatory and neuroprotective effects. In PD patients, elevated plasma levels of IL-13 have been observed, suggesting a pro-inflammatory state (Álvarez-Luquín et al., 2019). STAT3 in PD were upregulated in the striatum where it plays a role in activating inflammation pathway. While the specific mechanisms remain incompletely understood, emerging evidence indicates that STAT3 may influence several key molecules implicated in PD pathogenesis. STAT3 has been reported to activate microglia resulting in TNF-α expression (Lashgari et al., 2021). TP53 could potentially affect cell survival mechanisms linked to STAT3 signaling (Stechishin et al., 2013). STAT3 modulates PTGS2, which plays a critical role in mediating cell migration and inflammatory pathways (Lee et al., 2020). HIF1A and HSP90AA1 support cell function by regulating hypoxia adaptation and protein homeostasis (Lin et al., 2023; Xu et al., 2023). Although these genes do not directly interact with each other, they act synergistically through their common upstream regulator, STAT3, ultimately contributing to the anti-inflammatory and cell survival effects of the IL-4 and IL-13 signaling pathways. Therefore, we predict that the IL-4 and IL-13 signaling pathways are likely the primary biological effects influenced by *Cornus officinalis* in the treatment of PD.

Despite the strengths of bioinformatics platforms in identifying potential therapeutic targets and mechanisms, certain limitations must be acknowledged, including the frequency of data updates and the reliability of predictions. To address these limitations, future research should integrate experimental validation to substantiate the computational predictions. *In vitro*, we propose using PD-related cell models (e.g., MPTP-treated SH-SY5Y or PC12 cells) to evaluate the effects of *Cornus officinalis* compounds. Specifically, cell viability will be assessed using CCK-8 or MTT assays, oxidative stress will be measured via ROS detection, and apoptosis will be quantified through TUNEL staining. Additionally, the expression levels of key targets (HIF1A, PPARG, HSP90AA1, PTGS2) will be analyzed using Western blot or qPCR. *In vivo*, if the *in vitro* results are promising, PD mouse models will be employed to further validate the therapeutic potential of *Cornus officinalis*. Behavioral tests, including rotarod, open field, and hanging tests, will be conducted to assess motor coordination and activity. Histological and immunohistochemical analyses will quantify dopaminergic neurons and apoptotic markers in the substantia nigra and striatum. Furthermore, oxidative stress markers (e.g., MDA, SOD) and inflammatory factors (e.g., TNF-α, IL-1β) in brain tissues will be measured by ELISA, and the expression of key targets will be validated by Western blot or qPCR. Finally, to translate these findings into clinical practice, multicenter, large-sample clinical trials are essential to evaluate the efficacy and safety of *Cornus officinalis* in PD treatment. This comprehensive approach will bridge the gap between computational predictions and clinical applications, providing robust evidence for the therapeutic potential of *Cornus officinalis*.

## 5 Conclusion

Our study preliminarily elucidates potential targets and the pharmacological mechanisms of *Cornus officinalis* on PD through bioinformatics analysis. Additionally, molecular dynamics simulations were conducted to validate the binding stability of the active compounds of *Cornus officinalis* with their targets, further supporting their interactions. The results indicate that *Cornus officinalis* holds significant therapeutic promise as a potential therapeutic source for PD, likely mediated by achievingmultiple biological mechanisms, including anti-inflammatory actions, synaptic function improvement, mitophagy regulation, and neuroprotection. This research provides a theoretical foundation for further exploration of the mechanisms underlying *Cornus officinalis* in PD treatment.

## Data Availability

The original contributions presented in the study are included in the article/Supplementary Material, further inquiries can be directed to the corresponding author.

## Glossary

PD: Parkinson’s disease
PPI: protein-protein interaction
ROS: reactive oxygen species
GO: Gene Ontology
KEGG: Kyoto Encyclopedia of Genes and Genomes
TCMSP: traditional Chinese medicine systems pharmacology
TCM: traditional Chinese medicine
OB: oral bioavailability
ADME: Absorbsion Distribution Metabolism and Excretion
DL: drug-likeness
OMIM: Online Mendelian Inheritance in Man
MCC: Maximal Clique Centrality
TTD: Therapeutic Target Database
BP: biological process
CC: cellular components
MF: molecular functions
RMSD: Root Mean Square Deviation
RMSF: Root Mean Square Fluctuation
rGyr: radius of gyration
SASA: solvent-accessible surface area
EGFR: Epidermal Growth Factor Receptor
TP53: Tumor Protein p53
HIF1A: Hypoxia-Inducible Factor 1-Alpha
ESR1: Estrogen Receptor Alpha
PPARG: Peroxisome Proliferator-Activated Receptor Gamma
TNF: Tumor Necrosis Factor
HSP90AA1: Heat Shock Protein 90 Alpha Family Class A Member 1
PTGS2: Prostaglandin-Endoperoxide Synthase 2
MAPK1: Mitogen-Activated Protein Kinase 1
SRC: SRC Proto-Oncogene
α-syn: α-synuclein
