# Supplementary material for: Decoding the therapeutic potential mechanism of Cornus officinalis in Parkinson’s disease: a network pharmacology insight
Source: Front Pharmacol. 2025 Dec 1;16:1714796. doi: 10.3389/fphar.2025.1714796 (PMC12702959; doi:10.3389/fphar.2025.1714796)
Supplement: Supplementary file 1 [file Supplementaryfile1.zip › Supplementary Figure.docx]

**
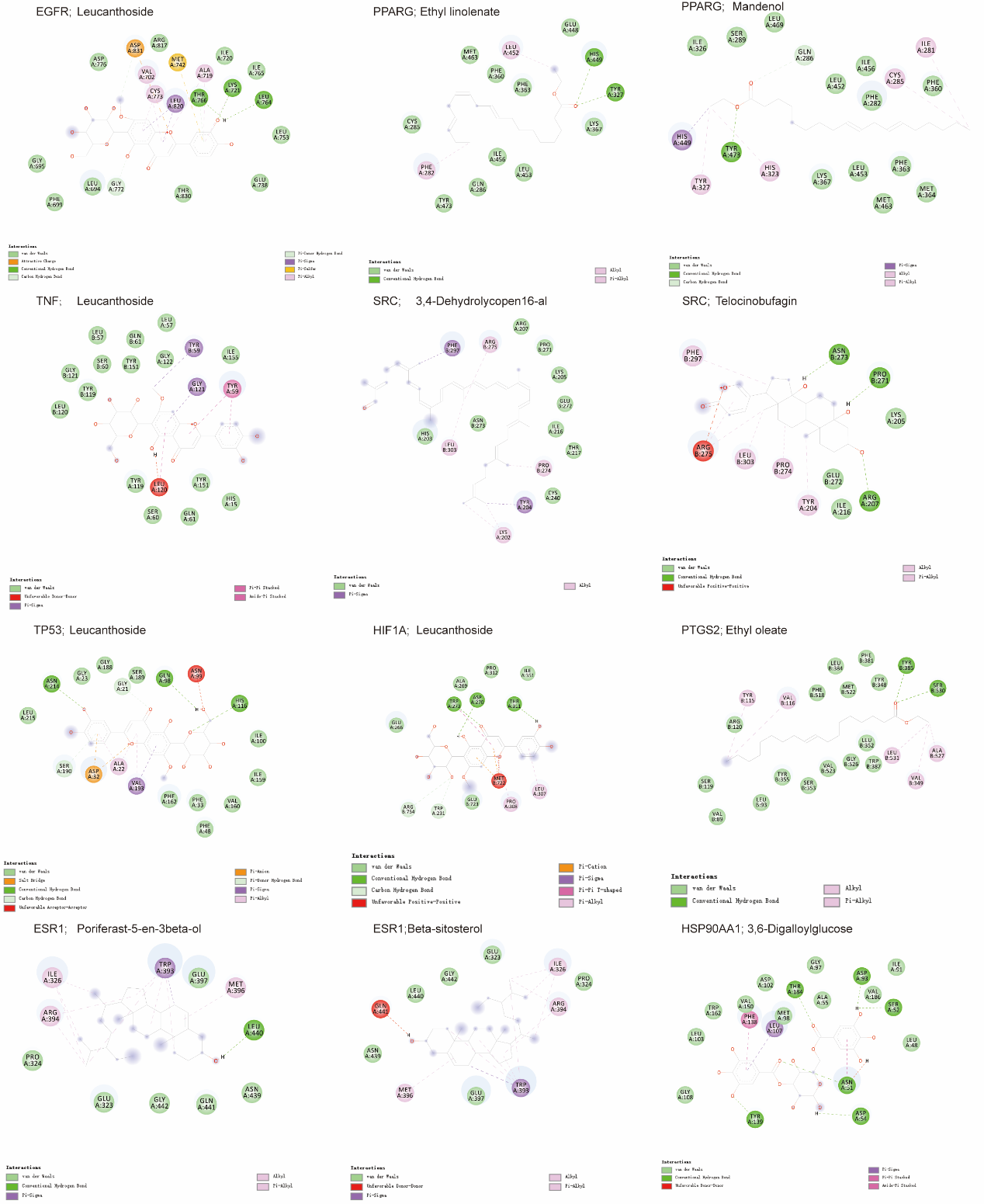
**

**Supplementary Figure 1. Diagrammatic 2D illustrated that the molecular docking model.**

**
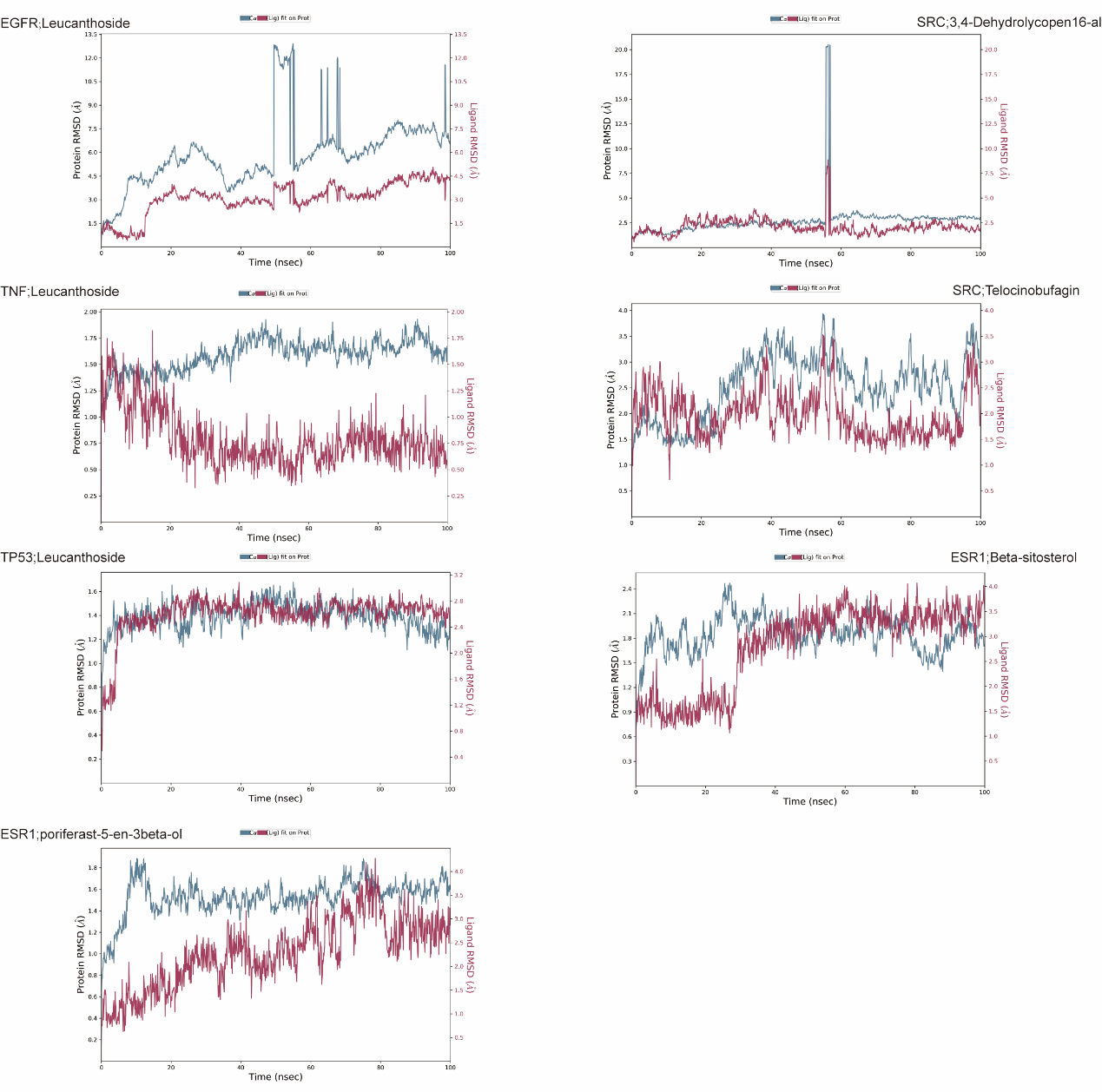
**

**Supplementary Figure 2. RMSD analysis of protein-ligand complexes.**

**
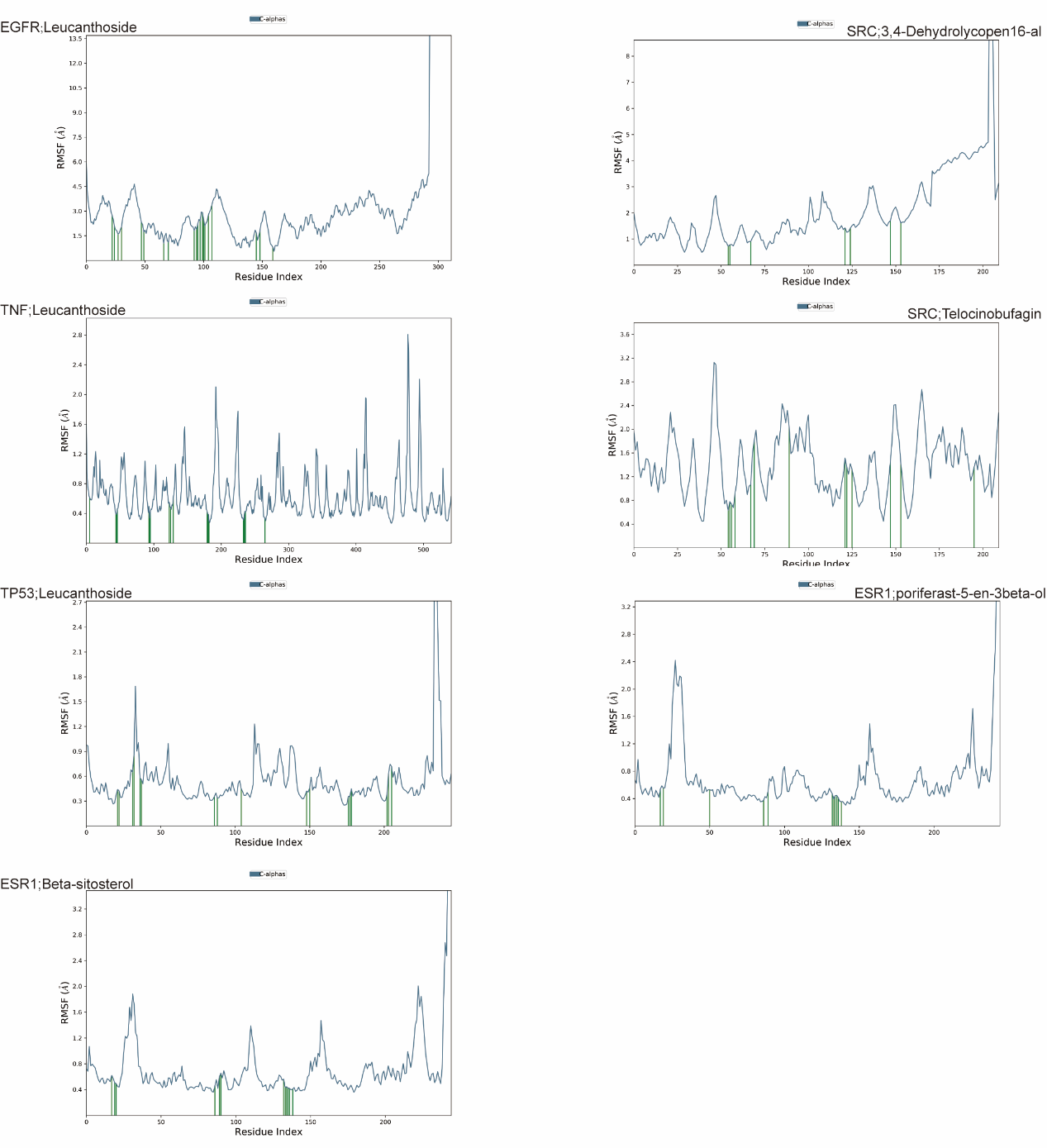
**

**Supplementary Figure 3. RMSF analysis of protein in complexes.**


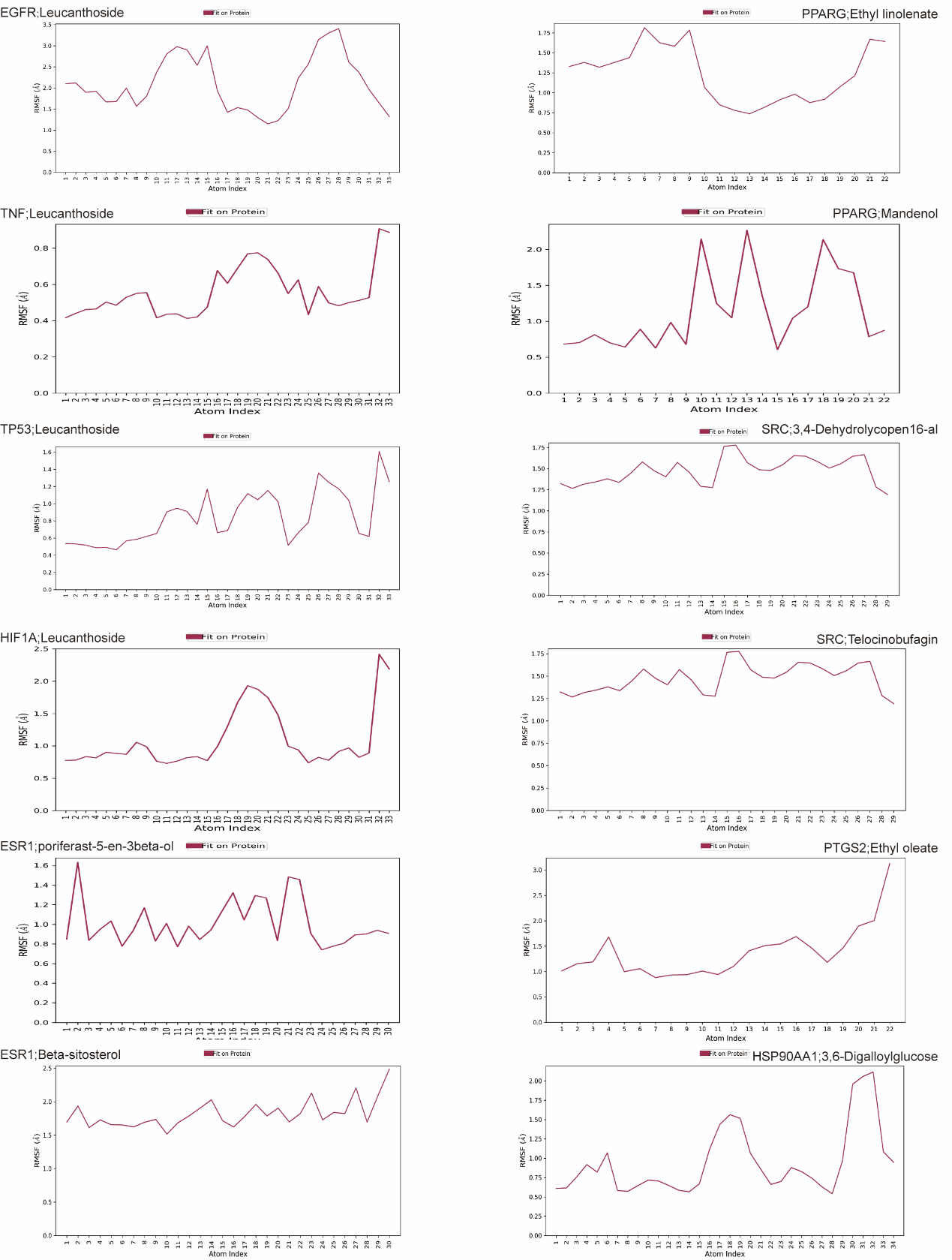


**Supplementary Figure 4. RMSF analysis of ligand in complexes.**

**
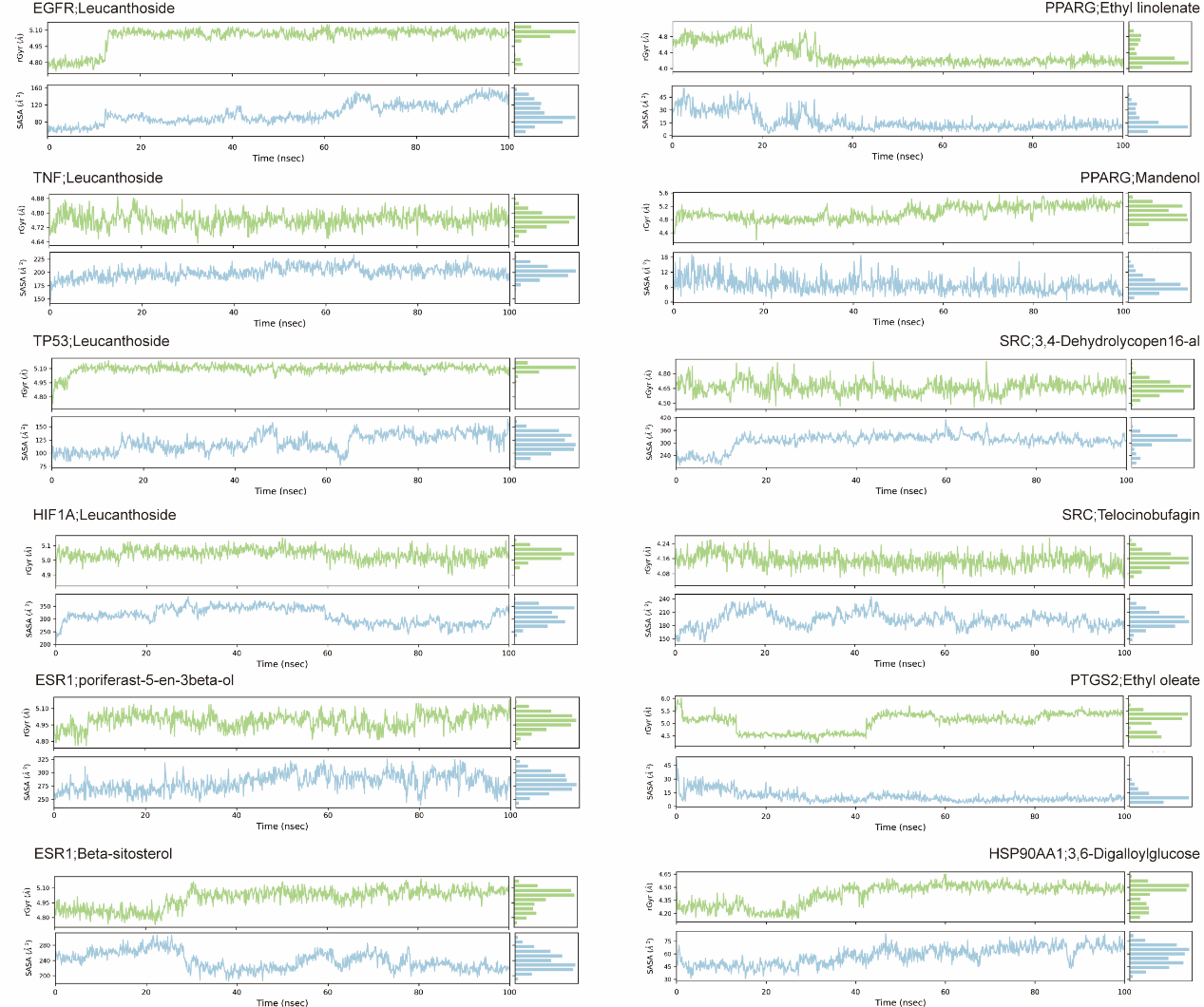
**

**Supplementary Figure 5. rGyr and SASA in molecular dynamics simulation of protein-ligand complexes.**
